# State Care of Epileptics

**Published:** 1896-03

**Authors:** 


### EDITORIAL DEPARTMENT.

F. E. DANIEL, M. D., Editor.
S. E. HUDSON, M. D., Managing Editor.
A. J. SMITH. M. D., Galveston, Associate Editor.

EDITORIAL STAFF:

PROF. J. E. THOMPSON, M. D., Texas Medical College, Galveston; Surgery.
PROF. WM. KEILLER, M. D., Texas Medical College, Galveston; Obstetrics and Gynecology.
PROF. DAVID CERNA, M. D., Texas Medical College, Galveston; Therapeutics.
PROF. A. J. SMITH, M. D., Texas Medical College, Galveston; Medicine.
DR. R. H. L. BIBB, Saltillo, Mexico; Foreign Correspondent.

Official organ of the West Texas Medical Association, the Houston District Medical Association, the Austin District Medical Society, the Galveston County Medical Society, and several others.

### STATE CARE OF EPILEPTICS.

The agitation of this reform, begun in Texas recently, or rather, lately revived, by Dr. H. A. Tutwiler in a letter to the TEXAS MEDICAL JOURNAL, and since urged by the JOURNAL, is beginning to show results. The Terrell Medical Society is the first organized body to take it up and discuss it. By reference to our pages, elsewhere, it will be seen that the proposition that the State shall take better care of the indigent of this class than is now being done, was well received. Dr. F. S. White, a skilled and experienced alienist,—a Texas physician, who has, with one exception, we believe, Dr. D. R. Wallace,—had a larger experience with the insane than any one in Texas,—introduced the subject, and in an address advocated the cause. The discussion culminated in the set of resolutions elsewhere printed.

The State has made munificent provision for the care of its insane, its deaf mutes and the blind; but extensive as it is, that for the insane has capacity for only about one-half the cases in the State; the others, of the indigent class, are *in jails.* This is a burning shame. Their sad condition should appeal to every humane man—doctor or not—in the State, and such pressure should be brought to bear upon the next legislature as to convince them that it is *their duty* to enlarge the asylum accommodations until every insane person in the State can be cared for;—and compel them to take immediate action, despite their perverted notions of economy, which lead them often to the most contemptible parsimony with regard to the State's funds. And, as the relation between the insane and epileptics is so close,—the two often co-existing,—no discrimination should be made in admitting them to the shelter of State care.

There is no excuse for not making provision for the better care of this class. The State is rich and has boundless resources; a very small addition to the present taxation—the addition of the fraction of a cent—would yield sufficient revenue to enable the authorities to care for the epileptics, in a manner more in accord with humanity than at present. All that is asked is, that the State shall put this class on a footing with other helpless unfortunates; for, as regards the expense of maintenance, that falls on the State under existing conditions. In the jails they have to be fed and clothed.

They should become wards of the State, and that care and protection given them that their helpless condition demands; and at the same time they should have the benefit of hygienic and medical treatment. Doubtless there are many whose miserable condition could be greatly relieved by it; and it is also more than probable, it is absolutely certain, that amongst them will be found cases amenable to surgical treatment. The traumatic cases, as a rule, are amenable to successful operation; and instances are recorded, in cases of unknown ætiology, where cures have followed castration.

Humanity demands that these people shall share the State's charity; that all shall be done that is possible to do, to relieve their miserable condition; and the first step is, to take them from the horrible, unwholesome, degrading environment of the jails, where they languish now, uncared for, on a level with the common criminal.

Dr. White's suggestion is a good one. The Craig colony for epileptics, lately established in New York, and now in successful operation, furnishes a model upon which to carry out his suggestions.

We are gratified to know that the Terrell Medical Society, through its committee, will bring the subject before the Texas State Medical Association at its annual meeting at Fort Worth, in April prox., and it is hoped and believed that that body will memorialize the legislature in the interest of this neglected class of unfortunates.

### A DISSENTER.

On the subject of State Care of Epileptics, the *S. W. Medical Record*, Houston, Texas, says: Just now some journals are giving considerable attention to a movement to get State aid for epileptics. These unfortunate cases, it is claimed, can be better taken care of and treated more skillfully when in care of the State than elsewhere. Just what the skillful treatment will be is not clearly outlined. At this writing, skillful treatment, with few exceptions, is either palliative or harmful. I suppose this State treatment will be to exhibit to each individual case a large number similarly effected, so that he will see how naughty it is to have a fit. By having an asylum for epileptics, the State will have an opportunity every two years to send back to the ranks a superintendent skilled as a specialist in diagnosing epileptic fits. He will be an authority in his section as to who should be sent to the asylum. Is it possible that the populists have disbanded and joined the ranks of the medical profession? Why not have State aid for syphilitics? Every subject is a menace to the public health, and in advanced stages truly are subjects of loathing and sympathy. Why not have the United States set apart one of our territories as an asylum for consumptives? They, too, are a menace to the public health, and in advanced stages truly helpless. Why not pension every man who can not make over $40 a month? Indeed, I think the advocates of this scheme are not reckoning upon the common sense of the public. R.

The above, coming from the source it does, and written by a representative physician of the better element of the Texas profession, we must say, strikes us with surprise. The profession of medicine is characterized by large sympathy; and so far as we know, without exception, it favors State care of its helpless, unfortunate ones. Who else is to care for them? Are not the insane, the deaf mutes, the blind and the paupers its wards and objects of its large charity? Why not, then, others equally as helpless, and more to be pitied, perhaps, than those named? And, indeed, is not the State now caring for them; if confining them in jails and feeding them on the rations furnished criminals can be called "caring for"; it is *bearing the expense of keeping them*, only, and it is this feature, it seems (by his reference to the populists) that Dr. Red objects to; he seems to be tinctured with the same kind of "economy" that characterized recent Texas legislatures, and feels that the better care of the epileptics by the State than they get in jail would be an extravagance which it is unreasonable to ask or expect, and ridicules the idea as absurd. I do not know what else his reference to populists can mean.

The border line between insanity and epilepsy is often so dim as not to be recognized, and distinction can only be made in extreme cases. Indeed, the two conditions very frequently co-exist —are merged; and hence, we have insane epileptics or epileptic insane. And as provision is made for the care and treatment of the insane, whether epileptic or not—why discriminate against the unmixed cases, a class just as worthy of our solicitude and charity. I apprehend that Dr. Red would not favor the abolition of the insane asylum, and relegate its inmates to the jails and poor houses, where they formerly were, and where the epileptics now are for the most part; but he seems willing that the epileptics shall stay there.

Dr. Red says:—"At this writing, skillful treatment, with few exceptions, is either palliative or harmful." (It would be interesting to know what "skillful treatment" was, the day before or the day after, that writing. Perhaps it has changed, and if not "palliative" is perhaps at least less "harmful.") Does Dr. Red mean to say that epilepsy is always incurable? or does the clause, "with few exceptions," cover the numerous cases of traumatic origin, and others of obscure causation, which have been cured by operation? The literature on the subject shows that many cases of epilepsy have been cured; indeed, about six per cent. of all cases. Dr. Blummer, in *American Journal of Insanity*, reports ten consecutive cases of epilepsy cured by castration. (This occurred, however, prior to "that writing.") At any rate, any kind of change from the present status of the unfortunates now in jail, would be a mercy.

It is not proposed to subject them to any "harmful" treatment, knowingly; and the treatment that is contemplated in advocating State care, is rational care, hygiene, clean beds, wholesome food, and more than all—protection; together with such healthful occupation as they are capable of performing, when not having paroxyms of "fits," of which affection the doctor speaks as if it were something greatly to be condemned,—a crime. and not a misfortune. He says, "I suppose this State treatment will be to exhibit to each individual case a large number of similarly effected [meaning "affected"], so that he will see how naughty it is to have a fit."—I dare say the poor creatures would gladly abstain from being "naughty" if they could. This sentiment does violence to both the head and heart of so excellent a physician as Dr. Red, and he is no doubt by now ashamed of having uttered it; we hope so at least.

The doctor appears to be impressed with the belief that those who are asking that the State shall do its duty alike by all its unfortunates without discrimination, and take this class out of jail and poor house, and put them on a footing with the other classes—do so on the ground that they are a "'menace to the public health";—and asks, why not include syphilitics and consumptives, as they are a menace *also?* It was thought hardly necessary to say,—the plea is based on humanitarian grounds, at the dictates of charity and mercy,—and not in the interest of public health; though when such cases of syphilis, or anything else, become, as the doctor says, "subjects of loathing and sympathy," they should be—if poor, and helpless, and dependent, objects of the State's tender care;—and in many States these find asylum in hospitals and infirmaries.

Dr. Red will find that the profession of Texas are not in sympathy with his views, and we can but regard his article as an error of judgment, and the attempt at jocularity as ill-timed and out of place. The sentiment is worthy of "Bell, of Cook," or Simpson, the senators who, when the venerable Cupples, and others of the Texas State Medical Association, were pleading for a law to regulate the practice of medicine,—moved to so amend as to embrace the barbers and the boot-blacks.

Dr. Red, as editor of a Texas medical journal, will be expected, if not to lead and shape the sentiment of the profession, to at least reflect it; and in this regard, we think, he will find himself lonesomely alone.

NEED FOR PROVISION OF THIS KIND.—Dr. Fred'k Peterson, President of the Board of Managers of Craig colony, and founder of the institution, in an article in *Pediatrics*, for February 1895, says:

"Epilepsy is a peculiar disease, characterized by loss of conscious ness and a convulsion. The fit of epileptic seizure occurs from time to time, and may last from a few seconds to a few miuutes, sometimes longer. Some patients have fits every day or oftener, some once a week, some once a month, some only once or twice a year. It is only during the fits that they are incapacitated. At other times they are well and strong and healthy looking, and quite as able to work and study as are other people. But the fact that they have these fits, no matter how rarely, debars them from many of the privileges enjoyed by their more fortunate brethren. They will not, on that account, be received into the public schools and can receive no education. They can not attend church or social gatherings. They are shunned by their playmates, and they become burdensome to their families. When they grow to adult life no one wishes to employ them, so, although they are able to learn a trade or profession, the shops and colleges are closed against them. No general hospital receives them as patients, and, in fact, there is no place at all which is open to them except an almshouse or an insane asylum, and as the insane asylum is better than the almshouse, many patients are sent there in preference to the poor-house."

Dr. Peterson says (*loc cit*): "A knowledge of the peculiar needs of epileptics gained by me during several years experience in one of our large asylums for insane, and a subsequent visit to the great colony for epileptics in Germany, (Bielefeld), which I described in the *N. Y. Medical Record*, in spring of 1887, led me to seek to establish in this country a colony of this kind, which should rival, if not surpass, its foreign prototype. To this end legislation was begun and carried on successfully until 1892, when the New York legislature passed a bill, empowering a commission to select a site for a colony for epileptics. Further legislation was required for its purchase and preparation for its benevolent purpose. It is now open and the first patients have been received there."

---

THE FORT WORTH MEETING of the Texas State Medical Association—its twenty-eighth annual meeting—to be held in the last week of April (prox.), beginning (4th) Tuesday, the 28th day, is looked forward to with unusual interest. A large attendance is confidently expected, as special rates over the roads centering at that point will be secured, an attractive programme and other inducements offered. Fort Worth is a central point, easily accessible from all parts of the State, and as the city has had a phenomenal growth, and is a curiosity in its way, there are many physicians who have never been there, who, it is expected, will take advantage of this occasion to visit the city. The fee for membership, too, will be, or has been, reduced one-half,—annual dues ditto; and as, in our judgment, this tax has heretofore operated, more than anything else, to keep membership down,—its reduction from $10 to $5 will have a revivifying effect. Let all the new made doctors, who have come into existence and into the State since last meeting,—and, in fact, all reputable doctors, new or old,—come; come into the fold, and help to push along the good work, begun many years ago. It received a check—a kind of paralytic stroke—from internal dissensions. The sorehead element has been eliminated and peace once more reigns. It is now in the convalescent stage. There was a kind of kiss and make-up at Dallas,—marred a little, it is true,—and for the first time in ten years or more, there was no knotty question of a personal nature, or of ethics, for the judicial committee to wrestle with; nor will there be at Fort Worth, it is hoped.

THE JOURNAL had expected to present the program, or at least a part of it, in this issue; but Secretary West writes us at a late day, saying, it will be impossible to get it ready, as the JOURNAL issues early in the month. It will appear complete, in the April number, which we will endeavor to get out far enough ahead of meeting time to disseminate the necessary information. Dr. Saunders, the Chairman of the Arrangement Committee, had not at the date of the secretary's letter sufficiently advanced his arrangements to enable the secretary to send us any announcements.

LET ALL WHO are preparing papers for this meeting,—and a general invitation is extended—to write upon such topics as may be thought of sufficient interest to bring before the Association, —remember that the paper—or at least the title of it, should be sent to the general secretary at as early date as possible, to insure a place on the program, and a time allotted for reading it. Papers reported too late to be announced on the printed program, have to take a back seat, and be read if opportunity occur; those listed, of course, having preference as to being read.

Once more, it is thought, a determined effort will be made to secure co-operation in the matter of medical legislation. Texas is far in the rear in this regard. Ohio has just succeeded, after as many refusals and setbacks as we have had, in getting through an excellent bill,—the Musgrave bill,—and the irregulars in that State will now have to "get further." They will pour into Texas, of course,—the dumping ground for all the refuse of other States. Thus a good law regulating the practice becomes an imperative necessity. It is a reproach to Texas that there are practically no restrictions on the privilege of practicing medicine in this State. Anybody can practice medicine in Texas; that is, if he have a diploma—*of any kind.* The writer, in his connection with the Public Health Department, is daily called upon to reply to inquiries as to the law, coming from physicians in other States;—letters addressed to the "Secretary of the State Board of Health,"—there being no such board and no such officer,—and we confess, it is most humiliating to have to tell them the *fact*, that *practically* there is *no* law on the subject.

The Ohio Profession obtained the law only after having organized, and each individual physician constituted himself a committee of one to work for the bill; and this is the way they worked—the way the "Red-Back" has advocated all the time: They went personally to each representative and senator, or to those expecting to be elected; and after having made them understand the situation and the necessity for the law, made their vote a condition, stipulating that the candidate should pledge himself to the passage of the Musgrave bill; swapped, as it were, vote for vote. And as there are seven thousand doctors in Ohio, seven thousand votes turned the scale; the candidates got in who had been "fixed" by the doctors, i. e., enlightened.

Now, in Texas, there are about five thousand doctors. If the Texas profession will do as the Ohio doctors did, and as the Journal advises, we can secure the passage of a medical act, not otherwise. And at Fort Worth such steps should be taken as are necessary to inaugurate such a campaign,—a "campaign of education."

The East Texas Medical Society have shaped up a bill, which, with some amendments, will do, and should be acted on at this meeting,—perfected, and sent to the next legislature by a committee.

